# Complementary feeding indicators from the World Health Organization and the Ministry of Health: agreement analysis and comparison of estimated prevalence in a cohort of children in southwestern Bahia, Brazil, 2018

**DOI:** 10.1590/S2237-96222024v33e2023556.en

**Published:** 2024-03-15

**Authors:** Clessiane de Brito Barbosa, Elma Izze da Silva Magalhães, Daniela da Silva Rocha

**Affiliations:** 1 Universidade Federal da Bahia, Instituto Multidisciplinar em Saúde, Vitória da Conquista, BA, Brazil; 2 Universidade Federal do Rio Grande do Sul, Programa de Pós-Graduação em Alimentação, Nutrição e Saúde, Porto Alegre, RS, Brazil

**Keywords:** Complementary Feeding, Infant Nutrition, World Health Organization, Cross-sectional studies, Alimentación Complementaria, Nutrición del Lactante, Organización Mundial de la Salud, Estudios Transversales, Alimentação Complementar, Nutrição do Lactente, Organização Mundial da Saúde, Estudos Transversais

## Abstract

**Objective:**

To assess the agreement between complementary feeding indicators established by the World Health Organization (WHO) and the Ministry of Health (MOH) and to compare the prevalence of these indicators in the first year of a child’s life.

**Methods:**

: This was a cross-sectional study in a cohort of 286 children from Vitória da Conquista, state of Bahia, Brazil; agreement between indicators and comparison between prevalences were analyzed using the Kappa coefficient and McNemar’s test; the prevalence of the indicators “introduction of complementary feeding” (ICF), “minimum dietary diversity” (MDD), “minimum meal frequency” (MMF) and “minimum acceptable diet” (MAD) were calculated.

**Results:**

: Three indicators showed poor agreement, with only one demonstrating moderate agreement; prevalence of WHO indicators was higher than that of the MOH (ICF, 94.3% vs. 20.7%; MDD, 75.2% vs. 50.7%; MMF, 97.2% vs. 44.8%; MAD, 96.8% vs. 26.9%).

**Conclusion:**

The majority of indicators showed poor agreement and the prevalence of WHO indicators exceeded that of the Ministry of Health.

## RESUMO

**Objetivo:**

Analisar a concordância entre indicadores de alimentação complementar da Organização Mundial da Saúde (OMS) e do Ministério da Saúde (MS) e comparar as prevalências entre esses indicadores em crianças no primeiro ano de vida.

**Métodos:**

Estudo transversal em uma coorte de 286 crianças de Vitória da Conquista, Bahia, Brasil; a concordância entre indicadores e a comparação entre prevalências foram analisadas pelo índice Kappa e teste de McNemar; foram calculadas as prevalências dos indicadores “introdução de alimentos complementares” (IAC), “diversidade mínima da dieta” (DMD), “frequência mínima de refeição” (FMR) e “dieta minimamente aceitável” (DMA).

**Resultados:**

Três indicadores apresentaram concordância ruim, e apenas um moderada; as prevalências dos indicadores da OMS foram superiores às do MS (IAC, 94,3% *versus* 20,7%; DMD, 75,2% *versus* 50,7%; FMR, 97,2% *versus* 44,8%; DMA, 96,8% *versus* 26,9%).

**Conclusão:**

A maioria dos indicadores tiveram concordância ruim e as prevalências de indicadores da OMS superaram as do MS.

## RESUMEN

**Objetivo:**

evaluar la concordancia entre indicadores de alimentación complementaria definidos por la Organización Mundial de la Salud (OMS) y el Ministerio de Salud (MS), y comparar la prevalencia entre estos indicadores en niños de un año.

**Métodos:**

estudio transversal en una cohorte de 286 niños de Vitória da Conquista, Bahía, Brasil; se calculó la prevalencia de “introducción de alimentos complementarios” (IAC), “diversidad dietética mínima” (DMD), “frecuencia mínima de comidas” (FMR) y “dieta mínima aceptable” (DMA); para evaluar la concordancia y comparar prevalencias se utilizó el índice Kappa y la prueba de McNemar.

**Resultados:**

cuatro indicadores mostraron un acuerdo pobre y sólo uno moderado; las prevalencias fueron mayores según la definición de la OMS (IAC, 94,3% vs 20,7%; DMD, 75,2% vs 50,7%; FMR, 97,2% vs 44,8%; DMA, 96,8% vs 26,9%).

**Conclusión:**

la mayoría de las concordancias entre los indicadores fueron deficientes, con prevalencias más altas según las definiciones de la OMS.

## INTRODUCTION

Indicators for assessing feeding practices have been developed to enable the measurement of aspects related to infant feeding in a simple, valid and reliable manner.^1^ The World Health Organization (WHO) has proposed several indicators for assessing the feeding practices in the first two years of a child’s life, which are frequently reviewed, updated, and used as instruments in the creation of useful reports for monitoring, evaluating, and directing actions and resources aimed at at-risk populations.^2^

The latest update of the WHO/UNICEF indicators, published in 2021,^3^ encompasses a set of 17 items related to continuous exclusive breastfeeding, feeding practices and bottle feeding. Nine of these indicators, specifically designed for evaluating complementary feeding in children aged 6 to 23 months, address dietary diversity, minimum meal frequency, specific nutrients, and consumption of unhealthy foods and beverages.^3^

At the national level, in 2015, the Brazilian Ministry of Health (MoH)^4^ published a guiding document for the assessment of food consumption markers, enabling the recognition of healthy or unhealthy feeding practices, in order to verify dietary patterns and facilitate the implementation of food and nutrition surveillance in the country.^5^ This document establishes 12 indicators for evaluating feeding practices in children under 2 years of age, including the introduction of complementary feeding, dietary diversity and minimum meal frequency.^4^

It is noteworthy that the indicators proposed by the WHO (2021) and those of the Ministry of Health (2015) establish minimum criteria to be met in infant feeding; however, the parameters considered for calculating indicators such as introduction of complementary feeding, minimum meal frequency, number and distribution of foods within food groups differ between both definitions. 3,4

Globally, numerous studies use complementary feeding indicators as parameters for assessing infant feeding.^5,6-11^ In Brazil, the most recent national survey on infant feeding, the Brazilian National Survey on Child Nutrition (*Estudo Nacional de Alimentação e Nutrição Infantil* - ENANI 2019),^12^ combined indicators proposed by the WHO (2021)^3^ with others created based on recommendations found in Brazilian dietary guidelines, in addition to indicators proposed by the Ministry of Health in 2015.^4^

Taking into consideration the scarcity of publications dedicated to a comparative analysis of these instruments,^13^ this investigation of the differences between the definitions from the WHO and the Ministry of Health, and their impact on the prevalence of adequacy of indicators, is justified. The aim is to guide the country’s health service management in choosing definitions that best represent reality and local feeding practices.

Thus, the objective of this study was to assess the agreement between complementary feeding indicators, as defined by the World Health Organization and the Ministry of Health, and to compare the prevalence of the respective indicators in the first year of a child’s life, in the municipality of Vitória da Conquista, state of Bahia, Brazil.

## METHODS

This was a cross-sectional study using data from the cohort study entitled “Breastfeeding and complementary feeding practice follow-up in children under 2 years of age living in the municipality of Vitória da Conquista – state of Bahia”. The municipality of Vitória da Conquista, located in the southwestern Bahia, had an estimated population of 343,643 inhabitants in 2021.^14^ Vitória da Conquista has four maternity hospitals: one of them provides care exclusively via the Brazilian National Health System (*Sistema Único de Saúde* – SUS); another provides only private care; and a further two provide both public and private care.

The cohort that gave rise to this study included puerperal women and their babies, living in the urban area and admitted to maternity hospitals in the municipality during childbirth. Participant recruitment took place between February and October 2017, initially through data collection in maternity hospitals, followed by home visits when the children were 30 days old and 6, 12 and 24 months old, concluding the follow-up in October 2019. More details about the cohort study that served as the basis for this work have been described in other publications.^15-17^ In this study, we used data collected during the follow-up of mothers and children at 6 and 12 months old.

Post hoc sample power was calculated, considering a 95% confidence interval (95%CI) and the prevalence of adequacy of each complementary feeding indicator (introduction of complementary feeding; minimum dietary diversity; minimum meal frequency; and minimum acceptable diet), for each estimated definition (WHO and MOH): six out of the eight prevalences considered for the calculation resulted in a power of 100%. Data collection at 6 and 12 months old was conducted through interviews, using the KoboTollbox 1.4.8® application, and data were digitized on tablets and smartphones with Android® operating systems. Trained researchers conducted the interviews to standardize data collection and prevent errors in the information obtained. In order to collect data on the children’s diet, an unvalidated structured questionnaire was used, containing questions about the consumption of 20 foods, related to various food groups (cereals, grains, roots and tubers; legumes; meat and eggs; vegetables; fruits; milk and dairy products). For each food, the mothers were asked if the child had consumed it the previous day and during which periods of the day (breakfast, mid-morning snack, lunch, afternoon snack, dinner, supper). The consistency with which the food was offered to the child was also asked (in pieces; mashed with a fork, leaving pieces; completely mashed with a fork; blended in a blender; sifted).

Information obtained on the food items consumed, meals taken and their consistency, on the day prior to the interview was used to construct variables for four selected indicators: (i) introduction of complementary feeding (ICF); (ii) minimum dietary diversity (MDD); (iii) minimum meal frequency (MMF); and (iv) minimum acceptable diet (MAD). The introduction of complementary feeding indicator was evaluated at 6 months old, while the remaining indicators were assessed at 12 months old. Definitions of these indicators, established by the WHO (2021)^3^ and the MoH (2015),^4^ along with the adaptations made, are shown in Box 1.

Statistical analysis was performed using Stata software version 14.0 (Stata Corp, College Station, Texas, USA). Agreement between indicators was assessed by calculating the Kappa coefficient. This measure ranges from a minimum value of 0 (zero), indicating no agreement, to a maximum value of 1 (one), representing perfect agreement. Kappa coefficient values below 0.20 indicate poor agreement; between 0.21 and 0.40, weak agreement; between 0.41 and 0.60, moderate agreement; between 0.61 and 0.80, good agreement; and between 0.81 and 1.0, excellent agreement.^18^ Differences between the prevalence of complementary feeding indicators, estimated according to the definitions from the WHO (2021)^3^ and the MoH (2015),^4^ was analyzed using the McNemar’s test. For all analyses, a significance level of 5% (p < 0.05) was considered.

The research project that gave rise to this work was approved by the Research Ethics Committee of the Instituto Multidisciplinar em Saúde da Universidade da Federal da Bahia (CEP-Seres Humanos/IMS/CAT/UFBA), in December 2016, under Certificate of Submission for Ethical Appraisal (*Certificado de Apresentação para Apreciação Ética* - CAAE) No. 62807516.2.0000.5556 and Protocol No. 1.861.163. Ethical aspects were followed in accordance with the National Health Council (*Conselho Nacional de Saúde* - CNS) Resolution No. 466, issued on December 12, 2012. All participating mothers signed the Free and Informed Consent Form.

## RESULTS

**Figure 1:**
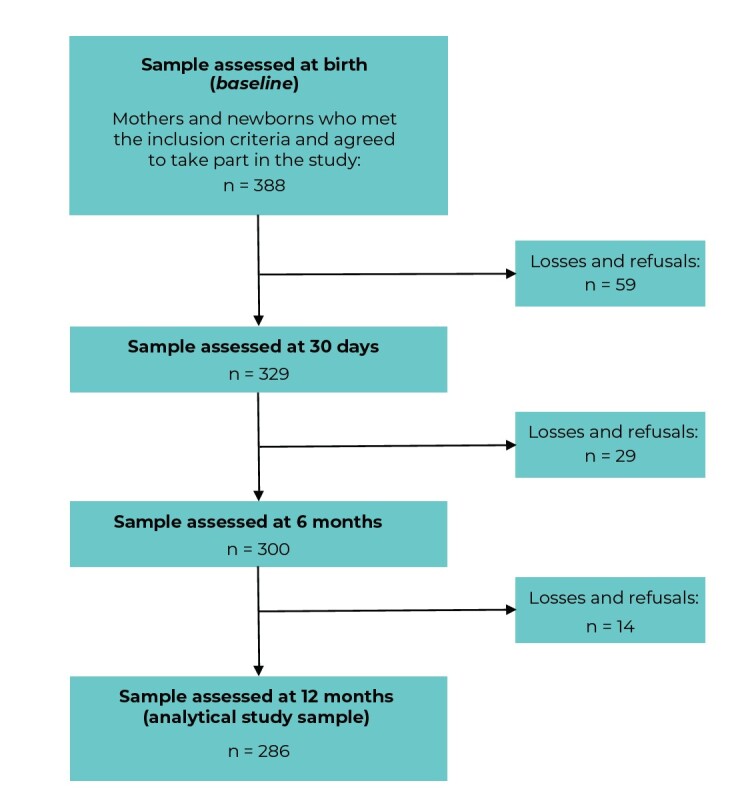
Flowchart of the sample of participants at each stage of the cohort studied and final sample (N = 286), Vitória da Conquista, state of Bahia, Brazil, 2018

Of the 388 mother-child binomials evaluated at the baseline of the cohort that gave rise to this study, 102 (26.3%) were lost to follow-up, totaling 286 binomials whose children were assessed at 12 months old and comprised the study sample (Figure 1). There were no statistically significant differences between the mother-child binomials at the baseline and those that constituted losses to follow-up (from maternity hospitals to 12 months), regarding the variables “family income” (p = 0.297), “paternal schooling” (p = 0.060), “maternal age” (p = 0.842) and “parity” (p = 0.285). As for the sample studied, 51.8% of the children were male, 52.8% were born by cesarean section and 3.5% had low birth weight. Most mothers were between 20 and 34 years of age (70.3%), had more than eight years of schooling (77.3%), and lived with a partner (77.3%); 74.0% of these women’s families lived with family income above one minimum wage.

With regard to the agreement analysis, the indicators of introduction of complementary feeding, minimum meal frequency and minimum acceptable diet showed poor agreement, with Kappa values well below 0.20: Kappa of 0.03, 0.05 and 0.01, respectively. However, for the minimum dietary diversity indicator, moderate agreement was observed: Kappa = 0.51. The minimum acceptable diet (MAD) indicator was the only one in which this agreement was not statistically significant: p = 0.139 (Table 1).

**Box 1:** Box 1 – Definitions of complementary feeding indicators, according to the World Health Organization (2021) and the Ministry of Health (2015), and adaptations made to the study in the first year of a child’s life, Vitória da Conquista, state of Bahia, Brazil, 2018

**Indicator**	**Definition**		**Adaptation made**
	**World Health Organization (2021)**	**Ministry of Health (2015)**	
Introduction of complementary feeding^a^	Percentage of children who consumed solid, semi-solid, or mashed foods on the previous day.	Percentage of children who consumed two fruits and one savory meal (cooked meal, porridge, or soup) on the previous day.	
Minimum dietary diversity^b^	Percentage of children who, on the previous day, consumed foods and drinks from at least five of the seven defined food groups: 1. breast milk; 2. grains, roots e tubers; 3. legumes; 4. dairy products; 5. meat; 6. eggs; and 7. fruits and vegetables.	Percentage of children who consumed the five related food groups the day before: 1. breast milk or milk other than breast milk; 2. fruits and vegetables; 3. meat and eggs; 4. beans; and 5. cereals and tubers.	The original definition from the World Health Organization, WHO (2021), required the consumption of at least five out of eight defined food groups, while the original definition from the Ministry of Health, MoH (2015), adopted the consumption of six related groups. For this study, the WHO definition (2021) did not include the food group “fruits and vegetables rich in vitamin A”; and the MoH (2015) definition did not include the food group “orange and dark green leafy vegetables or fruits”, due to the limitations of the food consumption questionnaire, which does not include specific types of fruits and vegetables.
Minimum meal frequency^b^	Percentage of children who, on the previous day, ate at least three meals with solid, semi-solid or pureed foods, in the case of breastfed children; or four meals with solid, semi-solid or pureed foods or dairy products (with at least one of the meals being solid, semi-solid or pureed), in the case of non-breastfed children.	Percentage of children who, on the previous day, ate savory food with a regular (in pieces) or pureed consistency at least twice a day.	
Minimum acceptable diet^b^	Percentage of children with a minimum dietary diversity and minimum meal frequency the previous day, according to WHO definitions (2021).	Percentage of children with a minimum dietary diversity and minimum meal frequency the previous day, according to MoH definitions (2015). Note: Although it is not an indicator listed in the document of indicators from this reference, it can be created from the indicators of minimum dietary diversity and minimum meal frequency, which are recommended by the MoH (2015).	

a) Assessed at 6 months of a child’s life; b) Assessed at 12 months of a child’s life.

**Table 1:** Agreement analysis among complementary feeding indicators in the first year of a child’s life (N = 286), Vitória da Conquista, state of Bahia, Brazil, 2018

Indicator	World Health Organization (2021) *versus* Ministry of Health (2015)			
	**Agreement** **(%)**	**Expected agreement (%)**	**Kappa**	**p-value**
Introduction of complementary feeding	26.3	24.0	0.03	0.015^a^
Minimum dietary diversity	75.5	50.4	0.51	< 0.000^a^
Minimum meal frequency	47.6	45.1	0.05	0.005^b^
Minimum acceptable diet	29.4	28.4	0.01	0.139

a) Kappa coefficient (p ≤ 0,05).

**Figure 2:**
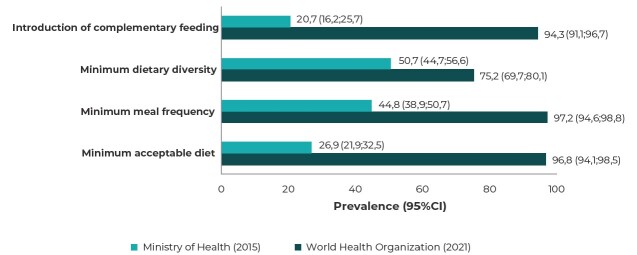
Comparison between the prevalence of complementary feeding indicators, according to the definitions from the World Health Organization (2021) and the Ministry of Health (2015), in the first year of a child’s life (N = 286), Vitória da Conquista, state of Bahia, Brazil, 2018

The description and comparison of the prevalence of the indicators, estimated according to the definitions from the WHO (2021) and the MoH (2015), is shown in Figure 2. The prevalence of the ICF, MDD, MMF, and MAD indicators, according to the definitions from the WHO and the MoH, were 94.3% vs. 20.7%, 75.2% vs. 50.7%, 97.2% vs. 44.8%, and 96.8% vs. 26.9%, respectively. The prevalence of the indicators assessed showed statistically significant differences between the two definitions (p < 0.05): higher prevalence was observed in the indicators based on the WHO definition, compared to the prevalence found based on the MoH definition.

## DISCUSSION

The results of this study demonstrated that three out of the four indicators assessed showed poor agreement between the definitions from the MoH and the WHO, as evidenced by differences in the estimated prevalence based on the corresponding indicators. The prevalence of all complementary feeding indicators was significantly higher according to the WHO definition.^3^ These findings suggest caution when choosing the definition for the indicators, which should take into account the differences between the definitions and their applicability in the local context.

The study showed poor agreement for most indicators, with the exception of the minimum dietary diversity, which was classified as having moderate agreement. However, the results of this analysis are useful in demonstrating that different instruments have the same ability to obtain identical results when applied to the same individual or phenomenon.^19^ It can be seen that the indicators defined by the WHO,^3^ compared to those defined by the MoH,^4^ despite having minimum parameters to be met in complementary feeding, are not able to measure the adequacy of feeding practices similarly in the population analyzed.

The introduction of complementary feeding was the indicator that showed the greatest discrepancy between the WHO prevalence (94.3%) and the MoH prevalence (20.7%). Data from the 2019 Brazilian National Survey on Child Nutrition (*Estudo Nacional de Alimentação e Nutrição Infantil* - ENANI),^12^ which used the WHO definition, the same as in 2021 and adopted in this cohort study, for the introduction of complementary feeding, found a prevalence of 86.3%. The differences between the definitions of this indicator may explain the results: while the WHO^3^ considers the consumption of any solid, semi-solid or pureed foods on the previous day as adequate, the MoH^4^ proposes, for the same indicator, the consumption of two fruits and a savory meal for 6-month-old children. It is noteworthy that offering any solid, semi-solid or pureed food without considering the nutritional quality of the food consumed may not represent an adequate food supply to the child at the time of introducing complementary feeding, as recommended by the current dietary guidelines for Brazilian children under 2 years of age.^20^

Although of lesser magnitude, the minimum dietary diversity also showed differences regarding the prevalence: 75.2%, according to the WHO definition,^3^ and 50.7% for MoH definition.^4^ The differences between the definitions of minimum dietary diversity lie in the fact that, for the MoH, breast milk and other dairy products account for a single food group, as well as a single group for meat and eggs, unlike the WHO definition, which classifies these foods into four distinct food groups, thus more easily reaching the minimum of five food groups required for dietary diversity.^3^,^4^

Meat in general, organ meats and eggs have similar nutritional characteristics because they are rich in protein, fat, iron, zinc and B vitamins, justifying their grouping as a single food group.^21^ Although breast milk is the main food in the group of milk and dairy products for feeding children under 2 years of age,^20^ other dairy products are also rich in protein, calcium and fat, justifying their grouping with breast milk.^21^

The indicator proposed by the MoH,^4^ in addition to dividing foods into food groups based on similar nutritional characteristics, as per national recommendations, establishes that all groups should be consumed by children to ensure a diversity of nutrients throughout the day.^20^ Therefore, the greater diversity of food groups in the diet is essential for maintaining adequate nutritional status and the formation of the child’s early eating habits. 20,22

The prevalence of the minimum meal frequency indicator according to the WHO definition^3^ was twice as high as the prevalence of the same indicator according to the MoH defiinition:^4^ 97.2% vs. 44.8%. The definition of this indicator, as proposed by the WHO,^3^ only takes into account the number of meals received, which can achieve adequacy with snacks as long as they are offered in solid/semi-solid/pureed consistency, thus overestimating their adequacy. On the other hand, the MoH definition^4^ is adapted to an eating routine, taking into consideration two main meals to achieve the minimum nutritionally adequate parameter.

The minimum acceptable diet indicator was the second with the greatest discrepancy in prevalence between the WHO and the MoH definitions: 96.8% versus 26.9 %. As this indicator is comprised of a combination of the minimum meal frequency and minimum dietary diversity indicators, the results reflect these other indicators.

Corroborating the findings of this study, a study conducted in Fortaleza, capital of the state of Ceará, in 2012, with data from 182 children at 15 months old from the Brazilian cohort of the MAL-ED study, also found high prevalence of the minimum dietary diversity (83.5%), minimum meal frequency (99.5%) and minimum acceptable diet (84.1%) indicators.^23^ using the indicators proposed by the WHO (2021).^3^

It is worth noting that in 2016, a study conducted with 1,355 children in the Southern region of the country already showed significant differences in the prevalence of complementary feeding indicators, when comparing the definitions previously proposed by the WHO in 2008 and by the Ministry of Health in 2010.^13^ In this sense, it is worth mentioning, based on the results of this study, that the differences in the definition of these indicators persist.

Furthermore, it is important to take into account that the WHO proposes general guidelines for assessing indicators, aiming to cover the widest possible variety of countries. However, each location has specificities to be considered when analyzing feeding practices. Studies show that local dietary guidelines are instruments that promote individual’s autonomy for healthy choices, and provide appropriate guidance for biological and sociocultural aspects, better reflecting the feeding practices of a society or country. 24,25

This study has limitations. The questionnaire for data collection on children’s feeding practices was not originally designed to estimate complementary feeding indicators, requiring adaptations to the original definitions of the minimum dietary diversity indicator established by the WHO^3^ and the MoH.^4^ As shown in Box 1, some food groups, both from the WHO and the MoH, could not be measured, taking into consideration that the list did not include the foods in these groups. Thus, caution should be exercised with the prevalence of minimum dietary diversity, which may be underestimated. However, considering that the adapted definition of minimum dietary diversity used in this study provides for the consumption of only five, instead of six, food groups, the prevalence estimate of this indicator established by the MoH^4^ may be overestimated. Consequently, the prevalence estimates of the minimum acceptable diet indicator by the different definitions may also be biased in the same direction.

Regarding the strengths, this study provided information on the types of foods consumed, their frequency of consumption and appropriate consistency, offering a detailed analysis of children’s feeding practices through the indicators constructed. In addition, it provides an important comparison between different definitions of national and international indicators.

In summary, the results show poor agreement among the majority of the indicators defined by the WHO and the MoH, reflecting differences found in the prevalence of the indicators, which are higher for the WHO indicators.

Although the Ministry of Health indicators are older, the criteria used for their definition are more detailed in assessing complementary feeding when compared to the definitions established by the World Health Organization, making their results more reliable in the scenario studied. Given the existence of national definitions, it is suggested that the evaluation of feeding practices should be guided by these, given their greater proximity to what is practiced and expected to be identified in the general population and, in the specific case of this article, in children under 2 years of age. Taking into consideration the limitation of the dietary assessment tool used in this study, further studies with instruments that include more specific foods, especially from the fruit and vegetable group, are recommended, enabling the estimation of more accurate prevalence.

## References

[1] World Health Organization (2008). Indicators for assessing infant and young child feeding practices: conclusions of a consensus meeting [Internet].

[2] Gatica-Domínguez G, Neves PAR, Barros AJD, Victora CG (2021). Complementary feeding practices in 80 low- and middle-income countries: prevalence of and socioeconomic inequalities in dietary diversity, meal frequency, and dietary adequacy. J Nutr.

[3] World Health Organization (2021). World Health Organization.

[4] Ministério da Saúde (BR) (2015). Secretaria de Atenção à Saúde.

[5] Pedraza DF, Santos EES (2021). Marcadores de consumo alimentar e contexto social de crianças menores de 5 anos de idade. Cad Saude Colet.

[6] Jones AD, Ickes SB, Smith LE, Mbuya MN, Chasekwa B, Heidkamp RA (2014). World Health Organization infant and young child feeding indicators and their associations with child anthropometry: a synthesis of recent findings. Matern Child Nutr.

[7] Silva VAAL, Caminha MFC, Silva SL, Serva VMSBD, Azevedo PTACC, Batista M (2019). Maternal breastfeeding: indicators and factors associated with exclusive breastfeeding in a subnormal urban cluster assisted by the Family Health Strategy. J Pediatr.

[8] Ortelan N, Neri DA, Benício MHA (2020). Práticas alimentares de lactentes brasileiros nascidos com baixo peso e fatores associados. Rev Saude Publica.

[9] Benvindo VV, Dutra AA, Menenguci MAS, Almeida NAV, Rodrigues AH, Cardoso PC (2019). Indicadores de saúde e nutrição de crianças menores de dois anos de idade: uma realidade para implantação da Estratégia Amamenta e Alimenta Brasil na atenção básica de Governador Valadares-MG. Demetra.

[10] González-Castell LD, Unar-Munguía M, Quezada-Sánchez AD, Bonvecchio-Arenas A, Rivera-Dommarco J (2020). Situación de las prácticas de lactancia materna y alimentación complementaria en México: resultados de la Ensanut 2018-19. Salud Publica Mex.

[11] Aryio O, Aderibigbe OR, Ojo TJ, Sturm B, Hensel O (2021). Determinants of appropriate complementary feeding practices among women with children aged 6-23 months in Iseyin, Nigeria. Scientific African.

[12] Universidade Federal do Rio de ﻿Janeiro﻿﻿ (2021). Universidade Federal do Rio de ﻿Janeiro﻿.

[13] Saldan PC, Venancio SI, Saldiva SRDM, Mello DF (2016). Proposal of indicators to evaluate complementary feeding based on World Health Organization indicators. Nurs Health Sci.

[14] Instituto Brasileiro de Geografia e Estatística (2021). Instituto Brasileiro de Geografia e Estatístic.

[15] Cirqueira RP, Novaes TG, Gomes AT, Bezerra VM, Pereira M, Rocha DS (2020). Prevalence and factors associated with tea consumption in the first month of life in a birth cohort in the Northeast Region of Brazil. Rev Bras Saude Mater Infant.

[16] Porto JP, Bezerra VM, Pereira M, Rocha DS (2021). Aleitamento materno exclusivo e introdução de alimentos ultraprocessados no primeiro ano de vida: estudo de coorte no sudoeste da Bahia, 2018. Epidemiol Serv Saude.

[17] Porto JP, Bezerra VM, Pereira M, Rocha DS (2022). Introdução de alimentos ultraprocessados e fatores associados em crianças menores de seis meses no sudoeste da Bahia, Brasil. Cien Saude Cole.

[18] Sim J, Wright CC (2005). The kappa statistic in reliability studies: use, interpretation, and sample size requirements. Phys The.

[19] Miot ﻿H﻿﻿A (2016). Análise de concordância em estudos clínicos e experimentais. J Vasc Bras.

[20] Ministério da Saúde (BR) (2019). Secretaria de atenção primária à saúde.

[21] Ministério da Saúde (BR) (2014). Secretaria de Atenção à Saú.

[22] Oliveira MIC, Rigotti RR, Boccolini CS (2017). Fatores associados à falta de diversidade alimentar no segundo semestre de vida. Cad Saude Colet.

[23] Andrade EDO, Rebouças AS, Quirino J, Ambikapathi R, Caufield LE, Lima AAM (2022). Evolution of infant feeding practices in children from 9 to 24 months, considering complementary feeding indicators and food processing: Results from the Brazilian cohort of the MAL-ED study. Matern Child Nu.

[24] Oliveira MSS, Santos LAS (2020). Guias alimentares para a população brasileira: uma análise a partir das dimensões culturais e sociais da alimentação. Cien Saude Col.

[25] Gabe KT, Jaime PC (2020). Práticas alimentares segundo o Guia alimentar para a população brasileira: fatores associados entre brasileiros adultos. Epidemiol Serv Saude.

